# Divided by a lack of common language? - a qualitative study exploring the use of language by health professionals treating back pain

**DOI:** 10.1186/1471-2474-10-123

**Published:** 2009-10-05

**Authors:** Karen L Barker, Margaret Reid, Catherine J Minns Lowe

**Affiliations:** 1 Physiotherapy Research Unit, BRU, University of Oxford & Nuffield Orthopaedic Centre NHS Trust, Oxford, OX3 7LD, UK; 2 School of Health Sciences, University of Birmingham, Birmingham, B15 2TT, UK; 3 Qualitative Researcher at Margaret Reid Research and Planning, Scotland, UK

## Abstract

**Background:**

The importance of using a common language when communicating to others about back pain is acknowledged in the literature. There are broadly three areas where difficulties in communication about back pain arise. Firstly, patients seeking information from health care professionals can experience difficulties understanding them and the medical literature; secondly, misunderstandings among health professionals concerning terminology can arise. Thirdly, the lack of standardised definitions for back pain terms can make comparison of research studies problematic. This study aims to explore the meanings and issues surrounding the use of existing medical terms for back pain from the perspective of health care professionals, lay people who have consulted health care practitioners for back pain and lay people who have not seen a health care professional regarding back pain.

**Methods:**

A series of focus groups were used to explore participants' understanding. A purposive sampling approach was used to achieve a sample which included general practitioners, chiropractors, osteopaths, physiotherapists, and lay people. Focus groups were facilitated by an independent professional qualitative researcher. They were audio taped and full transcripts of each focus group underwent line by line analysis, identifying concepts and coded. Constant comparison was used to allow each item to be checked or compared against the rest of the data

**Results:**

Lay participants understood the majority of the terms explored in the group differently to the health professionals. The terms, as understood by the lay participants, can be split into three broad categories. Firstly, terms which were not understood or were misconstrued and which had inadvertent negative connotations or implications. Secondly, terms which were not understood or were misconstrued, but without this leading to negative emotional responses. Thirdly, terms which were understood by lay participants as the health professionals stated they intended them to be understood.

**Conclusion:**

Few of the existing medical terms were understood and accepted by lay participants in the way discussed and expected by health professionals. Misunderstandings, unintended meanings and negative emotional responses to terms were common within the study focus groups.

## Background

Back pain is common. Two UK surveys report the lifetime prevalence of back pain as 59% [1,2] and 7% of the adult population consult with back pain in any one year [3]. Although common, the management of back pain is not straightforward and many patients continue to have long term back pain [4]. The importance of using a common language when communicating to others about back pain is acknowledged [5].

There are broadly three areas where difficulties in communication about back pain arise.

Firstly, patients seeking information from health care professionals can experience difficulties understanding them and the medical literature [5,6]. The use of jargon and of medical models rather than patient-centred lay models, together with poor communications skills from professionals are all considered contributing factors [6]. It is therefore important to shift from medical and scientific classification and terminology to a language understandable to lay people to develop a shared health care provider-patient language [5]. Furthermore, patients and professionals appear to define terms differently leading to misunderstandings when both think they are talking on common ground by using particular jargon [7,8]. Secondly, misunderstandings among health professionals concerning terminology can arise. Multiple back pain classification systems and terms exist and many health professionals use medical terms in various ways reflecting habit, experience, personal views and professional background [5]. This can hinder effective communication between professionals [9]. Thirdly, the lack of standardised definitions for back pain terms can make comparison of research studies problematic [10].

During a previous qualitative study exploring the views of lay people regarding the development and use of mass media interventions for health-care messages about back pain, it became apparent that further research was required to explore the issues surrounding the use of back pain language and terminology identified in the research [8]. Misunderstandings and unintended meanings for common back pain terms were observed. This study aims to explore the meanings and issues surrounding the use of existing terms regarding back pain, commonly used by health care professionals, lay people who have consulted health care practitioners for back pain and lay people who have not had back pain.

## Methods

### Sample

A purposive sampling approach was used to achieve a sampling frame which included general practitioners, chiropractors, osteopaths, physiotherapists and lay participants. (Table 1). The sampling frame for lay participants included men and women, a broad spread of socio economic groupings and ages and those with and without experience of back pain. All those with experience of back pain had sought professional help. Fifty percent of C2DE Socio Economic Group Members had manual occupations. Non English speakers were excluded. All health professionals worked in Birmingham or the West Midlands region and all lay participants lived in the West Midlands.

**Table 1:** Summary of groups

**Focus Group**	**Number of Participants**	**Details**
1. Chiropractors	N = 7 3 male, 4 female	Average experience of treating Spinal patients = 5.2 years Average spinal workload = 98.57% of total workload

2. General Practitioners	N = 8 6 male, 2 female	Average years in practice = 20.63 years

3. Osteopaths	N = 8 7 male, 1 female	Average experience of treating Spinal patients = 10.88 years Average spinal workload = 97.5% of total workload

4. Physiotherapists	N = 8 4 male, 4 female	Average experience of treating Spinal patients = 22.75 years Average spinal workload = 81.25% of total workload

5. Women	N = 7	C2DE, aged 20-39 No history of back pain

6. Men	N = 7	C2DE, aged 20-39 With a history of back pain

7. Women	N = 7	ABC1, aged 40-60 With a history of back pain

8. Men	N = 7	ABC1, aged 20-39 No history of back pain

9. *Men*	N = 7	C2DE, aged 40-60 With a history of back pain

### Focus Groups

Participants were approached and recruited by an independent professional qualitative research agency. Lay participants were approached on the street and were invited to a local focus group, which were held at convenient hotel venues. Health care professionals were approached by telephone and profession specific groups for Chiropractors, General Practitioners, Osteopaths and Physiotherapists were held, again at convenient hotel venues. Informed consent was sought at the groups. Participants were offered refreshments, and given either a £30 shopping voucher (lay participants) or £60 (health care professionals). Groups lasted for approximately one hour. All focus groups were lead by MR, an independent professional qualitative researcher. A list of medical terms was presented individually; verbally and also written on cards. The list was not intended to be exhaustive. Terms were identified from multiple sources including journals, textbooks and local copies of medical correspondence provided to patients. The list of terms was used in reverse order in four groups to minimise order effects. The Schedule can be seen in Table 2 and the terms in Table 3. Ethics approval for the study was sought and attained -REC reference number: 05/Q1605/62.

**Table 2:** Focus Group Schedule

**Groups**	**Questions used to facilitate focus groups**
**For all groups**	Do you think the language/type of words you use when you see your Patient/GP etc matters? Why? How? *For each term: *What do you think this word/phrase means? What do you think other people think it means (e.g. such as patients, GPs etc) What do you think having an (eg.Xray, MRI) will show? *Follow up prompts used to explore the meaning and understanding of each* *term*
**For Health Professional** **Groups only**	Are there any other words you used to describe back pain/ache to a patient? *Follow up prompts used to explore the meaning and understanding of each* *term*

**For Members of the** **Public Groups Only**	Are there any other words that you have heard used to describe back pain? What do you think (list of health care professions in turn) do when you have back pain? *Follow up prompts used to explore the meaning and understanding of each* *Term*.

**Table 3:** summarising the responses of members of the public to terms discussed in the focus groups.

**Speaking a different** **language - terms that could** **lead to problematic misunderstandings**	**Speaking a different** **language - terms with** **unintended meanings but** **few negative repercussions**	**Speaking a common language** **- terms which the public** **appeared to understand as intended**
Acute	(low) back pain/ache	Muscle spasm
Chronic	Mechanical back pain/ache	Sensation
Recurrent	Muscle sprain	Manipulation
Muscle Weakness	Muscle strain	Mobilisation
Instability	Sciatica	Soft tissue technique
Non-specific back pain	Radiated	Rehabilitation
Neurological involvement	Muscle imbalance	
Trapped nerve	Nerve root pain	
Paraesthesia	Disc - prolapsed, slipped,	
Managing your back pain	Herniated, ruptured	
Coping	Facet Joint	
Psychological pain	Alignment	
Wear and Tear	Posture	
Arthritis	Spondylitis	
Exercise	Stenosis	
Activity		
Disability		

### Data Analysis

Focus groups were tape recorded and full transcripts of each focus group underwent line by line analysis, identifying concepts and their properties. We used a two stage coding process common to qualitative enquiry [11]. Transcripts were listened to and read several times in order to become familiar with the accounts and transcripts were coded line by line (MR). This type of initial coding is important to theory development, but also helps the researcher remain close to the data and helps the researcher to challenge any a priori assumptions and to redefine categories based on what is found in the data. The second stage of data coding involves making a list of emerging themes and looking for connections between them. The aim of this second stage of coding is to make connections between segments of data and to develop theoretical concepts. Division between first and second stage coding is somewhat arbitrary. It might be more useful to think of analysis of the data, as an ongoing process that continually switches between first and second stage coding in order to remain close to the data, and to challenge any a priori assumptions. However, it is important to recognise that this process is an ***interpretative ***act and does not aim at scientific truth. The aim is to provide deeper understanding of human motivation and behaviour.

Triangulation proposes that by using multiple ways of looking at the same thing we can *converge *on the truth [12] The philosophical underpinning of this piece of qualitative research suggests that our aim is not to converge on reality, and that there will always be multiple perspectives (realities). In this study an independent researcher re-analysed the data on a line by line basis (CML) and another independent reviewer (KB) reviewed the analysis. Any differences of opinion were discussed and resolved. The aim of this was not to converge on a single truth, but to provide additional perspectives [13] In this study, member checking was not practical.

## Results

The results presented below focus on the findings we believe to be of most relevance to health professional readers.

### Common terms do have unintended meanings or negative connotations

Lay participants understood many of the terms explored in the group differently to the health professionals. The terms, as understood by the lay participants, can be split into three broad categories. Firstly, terms which were not understood or were misconstrued and which had inadvertent negative connotations or implications. Secondly, terms which were not understood or were misconstrued but without this leading to negative emotional responses. Thirdly, terms which were understood by lay participants as the health professionals stated they intended them to be understood. Although the lay participants who had been treated for back pain demonstrated some greater insight into some very specific terms, generally the areas and levels of understanding or misconception were similar to non back pain sufferers. The research demonstrated that familiarity with a term is no guarantee of understanding. At least one person in each group discussion with the lay participants attempted to define unfamiliar terms; usually by guessing. There is insufficient space here to present data for each term. We have chosen to focus on terms that were misunderstood by participants and had negative connotations or implications. Some of these are of particular interest as they are endorsed in low back pain clinical practice guidelines (acute, chronic, recurrent, disability).

### Non-specific back pain

No lay participants were familiar with the phrase non-specific back pain. For some it implied that the health professional did not understand the cause of their pain, or how to treat it. The phrase could suggest that the health professional thought that it was "*non-existent*". The phrase also has implications for treatment, suggesting that the patient would be automatically referred for further investigations or opinion; "*that spells referral to me*." Others felt that non-specific meant that pain is not located in a specific place; "*you would feel it all over really*", or that it could not be connected to a specific injury or habit.

Health professionals agreed that the intended meaning of non-specific was that a cause has not yet been found, or that there was no diagnosis. However, at the same time they recognised that patients needed to know that the health professional understood the cause of pain; the term suggested that the professional "*doesn't know what she's doing"*. They therefore said that they would not use the phrase to explain pain to patients.

*It's a posh way of saying I haven't got a clue ... it hurts our pride to put it down ... patients come to you because they want a diagnosis ... and you're not giving them one*.

### Acute

The lay participants were less familiar with the term *acute *than *chronic *in the context of low back pain. For some it suggested that pain was milder than chronic. For a minority of the low back pain sufferers, acute means recent or current. Acute was usually understood as severe, in a specific spot or sharp.

... *acute could be more localized ... its acute, just one spot. Like acute appendicitis. It's just that area*.

Health professionals believed that the intended usage of acute was pain of recent onset, but that lay people interpreted acute as a term which quantifies severity of pain.

... *It's a temporal thing rather than a qualitative*.

Health professionals therefore did not use the term to describe pain to patients, although they did use it in patient notes.

*I think patients if they go "Oh I've got acute back pain" they think it's like a quantifying factor ...that's why I don't tend to use it because ... it's incorrect language*.

### Chronic

The lay participants were very familiar with the term *chronic *in the context of low back pain. Most felt that chronic meant that the condition was very severe. To some is suggested that the pain was incurable.

*Chronic means absolute, the pits*.

*Couple of steps from a wheelchair*.

For others chronic meant long-term or constant

*(Chronic) lasts as well, doesn't it? Acute can be a short period of time*.

*I think chronic is long-term*.

Health professionals were aware that lay people interpreted chronic as severe and or "incurable", and preferred to use phrases such as "long-term", "long-standing" and "ongoing".

*It implies again something that's ... going to be there forever*.

*I don't use it that much because again it's like degenerative; it's unfair with "Is this it?" Am I going to be stuck with this?" It's scary*.

Some chiropractors and osteopaths thought that chronic might reassure someone that they would receive long-term treatment.

the only time I might use things like chronic is if somebody has had a condition for a very long time and they're wanting ... a diagnosis, then you can say this is a chronic condition and they can go "OK, now I can manage it"

Physiotherapists tended to write chronic in patient notes, but not say it to patients. They recognised that patients might interpret it in different ways.

*I think there are two definitions actually. One is comparing it with acute - in fact acute is like a thunderstorm if you like and chronic is like it's raining for a long time. But the patients, the elderly patients particularly often use the word chronic as something which is absolutely bloody awful*.

### Recurrent

In contrast to chronic, the term *recurrent *was interpreted by the lay participants as less severe and pain that comes and goes, whereas *chronic *pain never entirely going away.

*You've always got chronic. It comes in waves. But recurrent it can, chronic can't ever go down to zero; you've always got something wrong*.

GPs agreed that recurrent was often more accurate, useful and positive, than *chronic *in describing a patient's back pain.

... *I don't think that [chronic] is a word I really use for patients. They know that it's chronic ...I think recurrent is more of a description of it. Chronic implies that it goes on and on and will never go away*.

Physiotherapists did use the word *recurrent*, in particular in the context of the important educational role they have with patients.

*What I often tell patients ... our role ... is really to prevent recurrence by teaching the patient how to manage his back*.

### Muscle weakness

The lay participants were unfamiliar with the phrase muscle weakness in the context of low back pain. Several described it as a condition caused by inability to exercise or move the body; that *"muscles aren't exercised and giving adequate support*.

*If you were in plaster you wouldn't use the muscles in that particular leg. That would create muscle weakness*.

Some thought that muscle weakness was permanent and that it would progress.

Health professionals were concerned that patients felt muscle weakness was permanent, or that it implied personal weakness.

*I use weakness if you're talking about physio, muscle strengthening exercises and that sort of thing; not very often though ... but the weakness might imply permanent ... no I wouldn't go there*.

### Instability

Very few lay participants were familiar with the term instability. It was most often interpreted as the back could 'go' at any time.

*If they get you back to working order the back is unstable because the least little thing can actually throw it off again*.

*Something's a bit loose ... It's liable to pop out*.

Back instability was considered worrying. It tended to suggest a permanent condition and one from which a sufferer could never relax; "you're on a knife edge sort of thing.

*It is not in a stable state so it can't be localised and controlled. It can flare up at any time, there's not a lot you can do about it*.

Health professionals were aware of the negative connotations of the term and only used it in medical notes. Osteopaths used the words "loose" as an alternative, as they felt that this would not worry the patient as much. One osteopath said that the advantage of using a word that might worry a patient was that it would encourage the person to adhere to professional advice and maintain exercise regimes.

*They get a bit worried. I do use the word loose... It's a good way to almost, not scare the patients into it, but encourage patients to actually go and strengthen an area*.

Chiropractors tended to avoid instability seeing it as having the potential to cause alarm, and suggesting something more serious than it is.

*I think sometimes the word unstable, if you put that idea instability it might panic patients a bit but again it depends on the context and the patient ... I think they just assume it's perhaps worse than it is*.

### Neurological involvement

Both low back pain sufferers and non-sufferers rarely came across this term. A few understood it to have something to do with nerves or nerve endings. Most respondents immediately mentioned heads and brains; *"Something's going wrong in your head."*

Some thought that neurological referred to the brain and often pointed to the base of the skull while explaining it.

*Because it all stems from the brain doesn't it ... I think nerves because I think the brain ... your whole spinal cord runs up there doesn't it so your main nervous system runs up your back*.

Neurological involvement was one of the most alarming of the terms used. To some, it even suggested the possibility of imminent death.

*When your heads involved you start worrying don't you*.

*Death within six months*.

*Could be a tumour*.

Osteopaths, chiropractors and GPs said that they did not use the term neurological to explain pain to patients. This was not because they felt it might alarm the patient, but because it was not seen as useful without a diagnosis.

*It's not necessary. It doesn't add anything to the descriptors that we're already using. It's too vague really*.

Physiotherapists did use the phrase but stress that they always explained it, for example, in terms of loss of sensation. Neurological deficit was mentioned in the Physiotherapist group as preferable to involvement because it is more accurate.

### Trapped nerve

*Trapped nerve *was a very common phrase used by the lay participants, yet was poorly understood. Explanations were diverse. The lay participants described it as nerves stuck between bones or vertebral discs. For some this meant it was more serious, and for others, less serious. For some it involved 'inflammation'; for others, it meant no more than leading to pins and needles.

*I don't know because none of these things have ever been sat and explained. It's just things that you perceive yourself*.

Commonly, trapped nerve was interpreted as either the same as, or related to sciatica.

For health professionals, the term trapped nerve was introduced by patients rather than by themselves.

*It's one patients come in with a lot and you have to quantify to them exactly what a trapped nerve is to them so they don't use it again ... It's another misnomer really, a bit like a slipped disc*.

Health professionals were concerned that the term made the person's condition sound very serious and potentially untreatable or permanent.

*I don't (use it). I tend to say nerve root irritation. Again I think trapped nerve sounds a bit drastic and negative ... irritation's something that can be alleviated or eliminated*.

Some felt that trapped nerve can also be a 'catch all' cause of back pain, and sometimes they would have to correct a patient's understanding of it.

*You might correct it sometimes when people talk about ... having their trapped nerve which implies that it can't move and nothing can be done and you might just explain that it's probably a nerve that has some pressure on it and then discuss the different causes of pressure and some may be alleviated easily and some may not*.

Osteopaths discussed how the term trapped can mislead patients by suggesting that an expert must separate the bones to un-trap the nerve. GPs were concerned that trapped sounded unduly threatening and at the same time demanded detailed, lengthy and unnecessary explanation and so was avoided by some GPs although patients wanted to use it.

*I've often avoided the phrase trapped nerve because they want to know exactly what's trapping it and if it's serious*.

### Wear and tear

This was a commonly used and heard phrase for the lay participants in relation to low back pain. It was interpreted as the back "*wearing out" *or "*being worn out" *by age, work or sport. It was also described as "*general disintegration" *of discs or bones; part of the natural process of getting old. In the extreme it could be seen as meaning "*rotting away*".

*Wear and tear makes me think that something's actually diminishing. So, like a bone is getting thinner or a muscle is wearing thinner. It's shrinkage and it's unnatural. So that's what I think of wear and tear - something's rotting away*.

To the lay participants, this suggested there was no treatment and that you just have to live with it. "Degenerative change" was recognised as an alternative to wear and tear and was defined as progressively getting worse.

*No I wouldn't necessarily say age ... I think it might just be you've got one of those things. Like with arthritis it never gets better, it just gets worse*.

There was also a perception that nothing could be done to treat degenerative change. There were two very different emotional responses to a diagnosis of *wear and tear *Some were relieved that their pain was due to *wear and tear *and was not something "more serious."

*I'd feel relieved actually ... relieved it's nothing. I haven't got to have an operation, there's totally nothing wrong with me*.

However, the majority of lay participants said that a diagnosis of *wear and tear *would result in negative thoughts: I have to put up with this for the rest of my life as there is no treatment; it can only get worse; I am being fobbed off for wasting the doctor's time; I am disappointed to have no diagnosis; I am getting old before my time.

It's like they're taking the piss ... doctor sits there "Oh, it's just wear and tear."...It's quite condescending really isn't it when they say that? Especially when you're suffering

Osteopaths said that they used wear and tear rather than degeneration, thinking that it was kinder than arthritis or degeneration. Similarly Chiropractors and Physiotherapists use wear and tear though some insist it be accompanied by an explanation of how wear and tear or arthritis is not necessarily serious and that something can be done.

...*if you are describing wear and tear you must also say that's ok you've got this condition but we can do something to improve it*.

GPs did use the phrase but replace it with more medical or technical terms, such as degenerative spine disease, for 'sick notes' or medical notes.

### Arthritis

Many of the lay participants had never heard of arthritis in the context of back pain. Although the term was familiar in relation to other joints and in the elderly, knowledge of specific pathology is limited. Participants who had experienced back pain described it as: inflammation of the joints; bones rubbing together; crystallisation of the fluid in joints. Arthritis in backs was as particularly serious and worrying; "it means you're in big trouble". It was considered incurable, untreatable and progressive, and would be particularly concerning for young people.

*It'll get worse...you're diagnosed with that as you get older it's going to get worse and more painful ... There's not a lot of treatment for it that works*.

Health professionals were aware that *arthritis *could alarm patients, who might think that it was more serious than it often is.

We don't often use arthritis ... without specifically describing ... we have to go into a lot of detail to try and help people stop being anxious, because they come in terribly anxious, don't they?

Some physiotherapists use the term because their patients are likely to hear it from other professional, and took the opportunity to explain *arthritis a*nd any likely treatment for it. Most preferred to use the phrase *wear and tear*.

*Arthritis sounds like it's ill health and it's serious and a bit of wear and tear sounds just like living on this planet and normal and everybody in the room's got a bit. If somebody comes in and says they've got arthritis they're usually a bit more worried*.

Chiropractors similarly avoid *arthritis *in favour of *wear and tear *or *degeneration *as perceived to be less alarming.

*Oftentimes people don't want to hear they've got arthritis*.

...*Sometimes, people say the doctor's told me I've got arthritis and you say it's wear and tear it's normal ageing process they're quite relieved by that*.

Osteopaths criticised those who used the word *arthritis *without an investigative diagnosis, and did not to use the term.

*But they'll come away and I'll say "How do they know you've got arthritis?" "Oh, the doctor told me". "How does he know; did they do the blood test, did they do anything?" No, he just told me I had." ... they just tend to use it sometimes and we have to sometimes look at them and say well I don't see any arthritis I don't know where they've got that from*.

### Exercise and activity

For the lay participants, *exercise *and *activity *have distinct meanings and may infer these meanings upon the words used by professionals. *Exercise *is seen as a planned organised programme either for specific back strengthening or training or general working out at a gym for example. Whereas *activity *means day to day movement, moving about normally, such as walking.

*Exercise is always planned as opposed to activity (which is) just normal movement*.

*I think exercise is when you specifically go out to do like swim, go to the gym, workout. Whereas activity is your general activity during the day and how active you are*...

Some professionals, but not all, believe *exercise *to be similar to *activity *and use the terms interchangeably.

*You could use general exercise or general activity it means more or less the same thing*.

*There's prescribed exercise for your back and then there's exercises like walking and swimming but not jogging ... activity is anything...other than sitting in a chair or lying down ... activity implies normal lifestyle*.

(GPs)

See Table 3 for a summary for all the terms discussed and the categories into which the terms were placed.

## Discussion

Lay participants understood the majority of the terms explored in the group differently to the health professionals. The words discussed were often not understood or were misconstrued and many had inadvertent negative connotations or implications. Although aware of some of the ways in which patients may misunderstand words, the health professionals were unaware of some important effects words had on lay people. In this study, two different pictures of back pain language and consultation discussions emerged. The health professional groups showed awareness of the difficulties in communicating with back pain patients using language and reported their efforts and the strategies used to address them. The lay participants generally did not feel that health professionals adequately explained terms and back pain in an understandable way. The findings indicate difficulties exist in the communication between professional and lay perceptions and understanding regarding back pain language and identify the need for further work designed to address this gap.

### Strengths and limitations of the study

The sampling approach enabled us to select groups which purposefully informed our knowledge of our question area [14] and allowing us to explore a wide range of views for this early research. However, the study sample was drawn from English speakers from one geographical area and it is accepted that regional and language variations may exist and influence the findings. It is considered a strength of this study that a wide range of health professionals and lay participants were included. Since there is little previous research in this area we believed this breadth to be necessary to identify and raise relevant issues. However, this means that each professional group was represented by only one focus group one so the findings are not generalisable or representative of the professions involved in this research. Rather, the interesting differences and perspectives emerging regarding from the professional groups are considered as interesting questions to inform future work. Also, the professions in this study were chosen due to their high spinal workload and it is acknowledged that other professions have back pain patients and may use different terms and language.

Language is a component of the larger structure of communication but, whilst there are many studies emphasizing the importance of verbal behaviours for effective health professional-patient communication [15,16], there is little research directly assessing the importance of the language used. This may be because language is considered so personal, cultural, variable and non generalisable whilst key elements of communication, such as non verbal communication have wider applicability. The importance of specific language terms and the resulting connotations of words used for specific patient populations are however acknowledged [17]; Abramsky and Fletcher [17] concluded that the choice of words used to inform members of the public about prenatal diagnostic counselling may significantly affect how a genetic condition or risk is perceived. Cedraschi et al [7] demonstrated the presence of a discrepancy between the theory and practice of the word 'chronic' and the possible misunderstandings that the word could produce. They conclude, and this study agrees, that the widespread use of a word can erroneously encourage the belief that everyone is talking on common ground and attributing the same meaning. As Klaber Moffett et al. [18] opine, why do health professionals, who know the power of words to harm, help and promote change, pay so little attention to the actual words we use? The impact of simple changes, using long term rather than chronic for example, seemed evident in the lay member groups. Whether by increasing the awareness of health professionals regarding the understanding of existing terms the language gap can sufficiently be addressed, or whether health professionals and lay people need to co-develop new terms is an area of further research.

The importance and process of diagnosis was raised repeatedly within groups though never raised by the moderator. Health professionals acknowledged that, whilst patients are keen to receive diagnoses, most importantly they want treatment. Professionals appreciate that clear diagnoses and diagnostic labels are not possible for many back pain patients [5]. However, as indicated in this study, patients may believe a diagnosis is a prerequisite for effective treatment. Clear diagnoses are important; a recent review has concluded that greater attention should be paid to discussing diagnoses and causes of back pain with patients [19]. The lack of clear diagnoses and causal explanations can adversely affect patients; promote prolonged patient dependency [20], cause frustration [21] and impact upon patient compliance [5]. In this study, patients/former patients wanted clear diagnoses while professionals noticed patients returning to ask further "why, what"? questions and seeking information. Back pain is known to produce complex consultations; the lack of a definitive diagnostic test, fear of being labelled with psychological pain or as a malingerer, plus the need to provide proof of suffering can all contribute to difficulties from the patient perspective [20]. Whilst health professionals may wish to avoid conflict during consultations and find the application of recent research findings problematic [22]. It is also likely that health professionals and patients perceive and recall diagnosis and information sharing differently; our study supports McIntosh and Shaw's [21] qualitative study finding that patients report little evidence of receiving information from most health professionals although the latter all speak of providing such information. Barriers to information sharing clearly exist and finding the right words to use in health professional- patient consultations is a challenge [5].

In this study professionals continued to use words with patients which they appreciated were problematic. Either because the patient introduced them and they wished to respect the patient's language, because they lacked alternative terms or because they believed that using the terms with additional explanation surmounted the difficulties created by using a problem term. Ex-patients in this study did not offer any examples where such explanations overcame the effects of a poorly perceived term but this information was not specifically sought. While the health professionals in this study believed providing patients with information is integral to practice [21], a language gap seems evident. Health professionals should not assume that simple terms are necessarily better and should be aware that the use of a widespread term does not ensure patients and clinicians understanding it similarly. Commonly used terms may be misunderstood and have a negative impact upon patients. Furthermore there is a need for further dialogue between health professionals and patients regarding how present language gaps may be addressed.

## Conclusion

This preliminary research explores the clinically important, though scarcely researched, area of the language of back pain. Few of the existing medical terms included in this qualitative study were understood and accepted by lay participants in the way discussed and expected by health professionals. Examples of misunderstandings, unintended meanings and negative emotional responses to terms were found within the study focus groups. Health care professionals were sensitive and alert to the issues and reported the efforts they make to minimise potential problems caused by the presence of a language gap. As patient access to treatment notes and correspondence increases in the UK, the impact of written terms, as well as verbal, needs careful consideration and attention. Interesting issues have been identified that need further corroboration in future studies.

## Competing interests

The authors declare that they have no competing interests.

## Authors' contributions

KB led and designed the study and secured funding. She oversaw all data coding and analysis processes and independently reviewed data coding and analysis. She drafted the manuscript. CML checked all transcriptions, independently analysed and coded the data, and co-authored the manuscript. MR recruited the participants, conducted and transcribed the focus groups and performed the initial analysis for the study. All authors read and approved the final manuscript.

## Pre-publication history

The pre-publication history for this paper can be accessed here:


